# Efficacy and safety of ipratropium bromide/albuterol compared with albuterol in patients with moderate-to-severe asthma: a randomized controlled trial

**DOI:** 10.1186/s12890-016-0223-3

**Published:** 2016-04-30

**Authors:** James F. Donohue, Robert Wise, William W. Busse, Sandra Garfinkel, Valentina B. Zubek, Mo Ghafouri, Raymond C. Manuel, Rozsa Schlenker-Herceg, Eugene R. Bleecker

**Affiliations:** Division of Pulmonary Diseases & Critical Care Medicine, University of North Carolina, Chapel Hill, NC USA; Pulmonary and Critical Care Division, Johns Hopkins University School of Medicine, Baltimore, MD USA; Department of Medicine, University of Wisconsin, Wisconsin, WI USA; Boehringer Ingelheim Pharmaceuticals, Inc., Ridgefield, CT USA; Wake Forest School of Medicine, Center for Genomics and Personalized Medicine, Winston-Salem, NC 27157 USA; Previously of Boehringer Ingelheim Pharmaceuticals, Inc., Ridgefield, CT USA

**Keywords:** Randomized controlled trial, Moderate-to-severe asthma, Ipratropium bromide, Albuterol hydrofluoroalkaline, Ipratropium bromide/albuterol metered-dose inhaler, Anticholinergic/β_2_-agonist, Bronchodilation, As-needed, Acute symptom relief

## Abstract

**Background:**

Many patients with asthma require frequent rescue medication for acute symptoms despite appropriate controller therapies. Thus, determining the most effective relief regimen is important in the management of more severe asthma. This study’s objective was to evaluate whether ipratropium bromide/albuterol metered-dose inhaler (CVT-MDI) provides more effective acute relief of bronchospasm in moderate-to-severe asthma than albuterol hydrofluoroalkaline (ALB-HFA) alone after 4 weeks.

**Methods:**

In this double-blind, crossover study, patients who had been diagnosed with asthma for ≥1 year were randomized to two sequences of study medication “as needed” for symptom relief (1–7 day washout before second 4-week treatment period): CVT-MDI/ALB-HFA or ALB-HFA/CVT-MDI. On days 1 and 29 of each sequence, 6-hour serial spirometry was performed after administration of the study drug. Co-primary endpoints were FEV_1_ area under the curve (AUC_0–6_) and peak (post-dose) forced expiratory volume in 1 s (FEV_1_) response (change from test day baseline) after 4 weeks. The effects of “as needed” treatment with ALB-HFA/CVT-MDI were analyzed using mixed effect model repeated measures (MMRM).

**Results:**

A total of 226 patients, ≥18 years old, with inadequately controlled, moderate-to-severe asthma were randomized. The study met both co-primary endpoints demonstrating a statistically significant treatment benefit of CVT-MDI versus ALB-HFA. FEV_1_ AUC_0-6h_ response was 167 ml for ALB-HFA, 252 ml for CVT-MDI (*p* <0.0001); peak FEV_1_ response was 357 ml for ALB-HFA, 434 ml for CVT-MDI (*p* <0.0001). Adverse events were comparable across groups.

**Conclusions:**

CVT-MDI significantly improved acute bronchodilation over ALB-HFA alone after 4 weeks of “as-needed” use for symptom relief, with a similar safety profile. This suggests additive bronchodilator effects of β_2_-agonist and anticholinergic treatment in moderate-to-severe, symptomatic asthma.

**Trial registration:**

ClinicalTrials.gov No.: NCT00818454; Registered November 16, 2009.

**Electronic supplementary material:**

The online version of this article (doi:10.1186/s12890-016-0223-3) contains supplementary material, which is available to authorized users.

## Background

Despite the availability of effective asthma controller therapies, a significant proportion of patients have suboptimal asthma control, characterized by frequent symptoms, lifestyle restrictions, and healthcare use [1–3]. In addition to controller medications, acute reliever or rescue medications, e.g. short-acting β_2_-agonists (SABAs) are used to treat acute symptoms and exacerbations [4]. The frequency of SABA use as rescue medication reflects the frequency and intensity of symptoms, and is an important component for classifying asthma severity and level of disease control [4–6].

Short-acting anticholinergic agents have been used in asthma for decades; however, their exact role in asthma has not been well established [7, 8]. Studies directly comparing short-acting anticholinergic agents with a variety of SABAs have shown that SABAs provide greater bronchodilation than short-acting anticholinergic agents alone in stable asthma [9–11]; however, individual studies have demonstrated that specific asthma populations, e.g. older patients [12], and those whose asthma is related to psychogenic factors [13, 14], cigarette smoke, or β-blocking drugs [15, 16], might benefit from anticholinergic therapy.

Short-acting anticholinergic agents have also been evaluated in combination with a SABA or sequentially following SABA administration. The rationale for use of a combination of a short-acting anticholinergic agent and a SABA includes differences between the two classes of medications regarding mechanisms of action, side-effect profiles, onset and duration of action, and site of action [9]. Studies evaluating combination therapy with a short-acting anticholinergic and a SABA have shown variable results, mainly due to small numbers of patients or inappropriate patient populations [8, 11, 17–24]; however, many of these studies showed an additional (although not significant) benefit [8].

Combivent® inhalation aerosol metered-dose inhaler (CVT-MDI) is a fixed-dose combination of the short-acting anticholinergic, ipratropium bromide, and the SABA, albuterol sulfate, using a chlorofluorocarbon (CFC) propellant. It should be noted that the CFC-MDI formulation of Combivent (CVT-MDI) used in this study is no longer available, but Combivent is available in the Respimat® Soft Mist™ inhaler (Combivent® Respimat®), which is considered therapeutically equivalent to the CFC-MDI formulation, and studies in chronic obstructive pulmonary disease (COPD) have indicated that it has similar bronchodilator effects [25, 26].

A prior, single-dose, double-blind, crossover study compared CVT-MDI to albuterol hydrofluoroalkaline (ALB-HFA) in patients with moderate-to-severe asthma and persistent symptoms. These patients required regular use of albuterol as rescue medication (6–56 puffs per week) despite medium- to high-dose inhaled corticosteroids (ICS), with or without long-acting β_2_-agonist (LABA) [22]. Patients demonstrated significant improvement in pulmonary function after treatment with CVT-MDI versus ALB-HFA alone. We designed a study based on these results, and according to results from the Asthma Clinical Research Network studies [27], which indicated that inhaled long-acting muscarinic antagonists (LAMAs) might be an alternative to LABAs and other controllers in patients whose condition is inadequately controlled on an ICS alone [28].

The objective of this study was to evaluate whether CVT-MDI provides more effective acute relief of bronchospasm in moderate-to-severe asthma than ALB-HFA alone after 4 weeks, as add-on to stable doses of their controller medications (ICS, LABA, leukotriene modifier, theophylline, anti IgE, oral corticosteroids). Patients were required to be on stable doses of these medications for at least 4 weeks prior to screening to achieve a clear baseline before study commencement. The efficacy of CVT-MDI or ALB-HFA was compared for acute improvement in lung function at the beginning and end of treatment, and other measures of asthma control (medication was withheld before measurement of acute response). Patients were instructed to use open-label ALB-HFA in addition to the blinded study medication if required.

## Methods

### Ethics, consent and permissions

This randomized, double-blind, two-way crossover study was conducted in accordance with the Declaration of Helsinki. The protocol and all amendments were approved by the institutional review board/ethics committee at each participating site (Additional file 1: Table S1). All patients provided written informed consent.

### Trial design

Every effort was made to collect spirometry data for each time-point at the clinic visit. To be able to include the same patients at each time-point in the spirometry summaries, missing values were estimated using other values recorded for the patient on that test day. For patients with missing data on a given test-day because additional SABA medication was taken during testing, missing data were estimated by the least favorable observation on that test-day. Randomly missing data were estimated by either linear interpolation of adjacent data or by the last observed data if no subsequent data were available. The decision to use estimates for missing data was made prior to unblinding of the treatment assignments.

### Participants

Patients were male or female, ≥18 years of age, with physician diagnosis of asthma for ≥1 year, baseline forced expiratory volume (FEV_1_) ≤80 % predicted normal and post-bronchodilator reversibility of ≥12 % or ≥200 ml after administration of four puffs of ALB-HFA. Spirometry was performed according to American Thoracic Society guidelines [29, 30], and National Health and Nutrition Examination Survey reference equations were used to calculate predicted values [31]. Patients received treatment with ICS with or without LABA and other asthma controller medications for ≥6 weeks prior to screening, and used a short-acting bronchodilator ≥3 times a week for symptom relief in the 2 weeks prior to screening. Patients were entered in the study only if they had an Asthma Control Questionnaire (ACQ) score of ≥1.5 [32].

Patients were excluded from the study if they had been diagnosed with COPD or other significant disease; however, all patients who were non-smokers or ex-smokers who stopped smoking for >1 year prior to study participation and had a smoking history <10 pack-years were eligible. Patients who had been hospitalized for cardiac failure in the past year or who had a recent history of myocardial infarction were excluded.

The study included a 2-week screening period to establish patients’ baseline asthma measures and confirm eligibility using bronchodilator reversibility testing—12 % and 200 ml improvement in FEV_1_ post-bronchodilator after four puffs of ALB-HFA MDI—at visit two. Patients recorded symptoms, medication use (maintenance ICS ± LABA and “as needed”) and peak expiratory flow (PEF) in an electronic diary (eDiary)/peak flow meter (Asthma Monitor® [AM3]; ERT Products, Philadelphia) during the screening period. After the run-in period, eligible patients were randomly assigned (1:1) to either CVT-MDI or ALB-HFA. Patients used blinded study medication as needed (two puffs every 4–6 h, up to four times daily) between visits for symptom relief during the 4-week treatment period. Additionally, patients received open-label ALB-HFA for use if symptom relief could not be achieved with the blinded study medication. Maintenance therapy with high- or low-dose ICS had no impact on outcomes (Table 2) and, as this was a crossover study, all patients served as their own control.

Following the first 4-week treatment period, patients had a 1–7 day washout period (a 6–8-hour wash-out period is generally considered adequate for short-acting antimuscarinics (SAMAs) before entering the second 4-week treatment period with crossover treatment using either CVT-MDI or ALB-HFA. All other (non-asthma) concomitant therapies taken at screening and throughout the trial period were recorded. For washout, patients were instructed to refrain from using their study medication for at least 6 h prior to the scheduled clinic visit. On days 1 and 29, patients underwent lung function testing with 6-hour serial spirometry. Baseline FEV_1_ was measured 10-minutes before, and at 5, 15, 30, 60 min, and 2, 3, 4, 5, 6 h after study drug administration.

Patients were instructed to use the asthma monitor (AM3) throughout the study. Patients used the AM3 to record twice-daily peak expiratory flow (PEFs), as-needed study medication use, additional open-label ALB-HFA use, daily symptom assessments, and background controller medication use (such as ICS, LABA, leukotriene modifier, theophylline, anti IgE, OCS) for the duration of the study.

### Interventions

CVT-MDI (Combivent® Inhalation Aerosol CFC-MDI (Boehringer Ingelheim Pharmaceuticals, Inc., Ridgefield CT, USA) or ALB-HFA (IVAX Pharmaceuticals, Waterford, Ireland) was used during clinic visit days for pulmonary function testing, and as needed between clinic visits for symptom relief. Each actuation of CVT-MDI delivered 18 μg ipratropium bromide and 103 μg albuterol sulfate (equivalent to 90 μg albuterol base) from the mouthpiece. For the ALB-HFA MDI, each actuation delivered 120 μg albuterol sulfate from the canister valve and 108 μg albuterol sulfate from the actuator mouthpiece (equivalent to 90 μg albuterol base). During each treatment period, patients recorded in their eDiary the number of puffs of study medication taken (AM and PM).

If patients perceived that study medication was not adequately controlling their asthma symptoms, they were instructed to use the open-label ALB-HFA (ProAir® HFA, IVAX Pharmaceuticals, Waterford, Ireland) in addition to the study medication.

For the study duration, patients were required to remain on stable doses of their asthma controller medications and changes in background controller medications were documented. Additions or increases in the dose of oral corticosteroids were allowed for the management of asthma exacerbations and were recorded.

### Study end points

Co-primary endpoints were FEV_1_ area-under-the-curve (AUC_0–6_) above test-day baseline from 0 to 6 h, and peak FEV_1_ response. The study defined peak FEV_1_ response as the maximum change in FEV_1_ from test-day baseline within the 6-hour post-treatment interval, after 4 weeks of treatment.

The secondary endpoints were the mini Asthma Quality of Life Questionnaire (mini-AQLQ) responses, ACQ-7 responses, and the number of puffs of study medication and open-label ALB-HFA (AM and PM) patients used during each treatment period. Other endpoints included forced vital capacity, peak expiratory flow (data not shown), night-time awakenings due to asthma symptoms (from eDiary), and duration of bronchodilator FEV_1_ response. Bronchodilator response was achieved if an FEV_1_ value of ≥1.15 times the corresponding test-day baseline value was recorded at any time-point during the first 6 h after treatment administration. Termination of bronchodilator response was identified by the first fall of <1.15 times the corresponding test-day FEV_1_ baseline value on two consecutive measurements following a bronchodilator response during the 6-hour observation period on the test day. Duration of bronchodilator response was defined as the time interval between onset (bronchodilator response first achieved) and termination of bronchodilator response; duration was zero if there was no bronchodilator response.

Safety endpoints included monitoring of adverse events (AEs), vital signs, screening laboratory values (hematology, chemistry, and urinalysis), and electrocardiogram (ECG).

### Statistical methods

Co-primary efficacy endpoints of CVT-MDI and ALB-HFA were compared using mixed-effect model repeated measures (MMRM). The MMRM model has treatment, period, day, and the interaction between treatment and day as fixed effects, test-day baseline value as a covariate, and patient as random effect. In the MMRM model, day refers to the start (day 1) or end (day 29) of 4 weeks of treatment. In the results section, only results for day 29 will be presented. A hierarchical testing procedure was used to address multiple comparisons. The null hypothesis for FEV_1_ AUC_0–6_ response was tested first (alpha = 0.025, one-sided); if this null hypothesis was rejected, the null hypothesis for peak FEV_1_ response was tested next (alpha = 0.025, one-sided).

The primary efficacy analysis was performed on a modified full analysis set (FAS). The FAS consisted of all patients receiving study medication, who were documented to have taken at least one dose of investigational drug, and who had no missing test-day baseline values or missing responses for FEV_1_ AUC_0–6_ and peak FEV_1_ after 4 weeks of treatment. Seven patients who reported that their study medication devices were working improperly, and whose treatment blind was broken prior to database lock, were excluded from all efficacy analyses.

A post-hoc analysis was also performed on endpoints related to the co-primary endpoints. The MMRM model described above was used to analyze the ratio of FEV_1_ AUC_0–6_ response to test-day baseline FEV_1,_ and the ratio of peak FEV_1_ response to test-day baseline FEV_1_.

Subgroup analyses for the co-primary endpoints were performed for: onset of asthma; type of asthma; percent predicted FEV_1_ categories; concomitant asthma medication usage; puff usage of medication at study baseline; gender; race; age categories; smoking status; and FEV_1_/forced vital capacity (FVC) percentage categories based on pre-bronchodilator measurements at randomization. Subgroup analyses were performed to test whether the treatment effect was uniform across subgroups, at the end of the 4-week treatment period.

Secondary endpoints were analyzed using the MMRM model described for the primary analysis. The analysis of the duration of bronchodilator response was pre-specified as descriptive statistics; a post-hoc analysis of bronchodilator response duration on day 29 was also performed using an MMRM model with treatment and period as fixed effects, and patient as random effect.

A post-hoc responder analysis (McNemar’s sign test) was performed to test for the difference in the proportion of responders (those who achieved bronchodilator FEV_1_ response) between treatment groups.

All safety data were displayed and analyzed using descriptive statistical methods.

Based on a recent, single-dose, crossover trial with CVT-MDI and ALB-HFA, the standard deviation for the difference between treatment groups (paired t-test) in FEV_1_ AUC_0–6_ was expected to be 200–220 ml [22]. For the peak FEV_1_ endpoint, the mean difference between treatments and the standard deviation of the mean treatment difference were expected to be similar to that observed for FEV_1_ AUC_0–6_ (usually AUC_0–3_ is similar to peak FEV_1_ – AUC_0–6_ also includes duration of effect of these relatively short-acting bronchodilators).

To detect a 60 ml difference in mean values (based on the single-dose crossover trial) using a 2.5 % level of significance (one-tailed) and 90 % power, a sample of approximately 144 completed patients was calculated; this was increased to 200 to adjust for patients who dropped out prior to completing both 4-week periods. With an estimated 15 % discontinuation rate, 170 patients were expected to complete the first and second periods of the crossover (phases I and II).

## Results

**Fig. 1:**
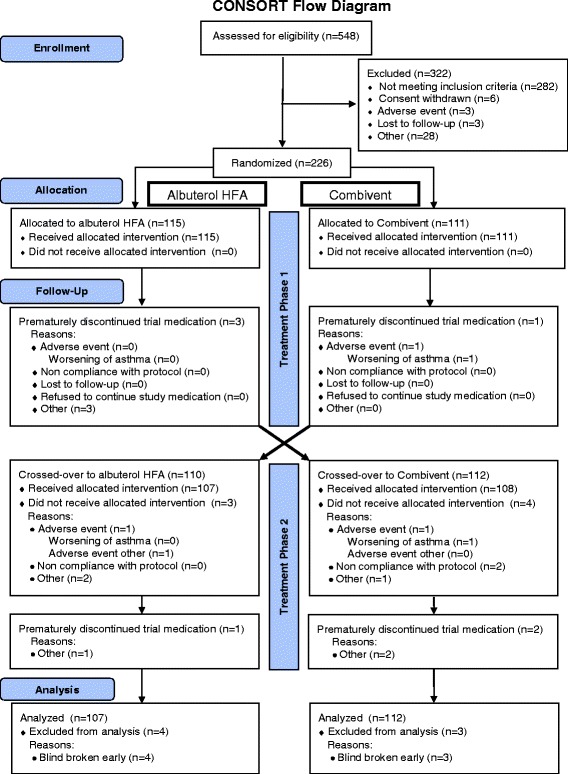
Consolidated Standards of Reporting Trials (CONSORT) flowchart of patient disposition

A total of 548 patients recruited from 41 study centers in the United States from December 2008 to September 2009 were screened and 226 patients were randomized using a validated system and received at least one dose of study medication; 222 randomized patients were treated with ALB-HFA, and 219 with CVT-MDI (107 received ALB-HFA, then CVT-MDI and 112 received CVT-MDI, then ALB-HFA). During the crossover period, 14 patients prematurely discontinued study medication; three patients had AEs leading to discontinuation (one taking ALB-HFA, two taking CVT-MDI), and only 3 % of patients had missing data. Patient disposition is presented in Fig. 1, and demographics and baseline characteristics in Table 1.

**Table 1:** Summary of demographic and baseline characteristics of randomized patients

Total No. of randomized patients, no (%)		226 (100)
Female, no. (%)		130 (57.5)
Race, no. (%)	White	174 (77)
	Black African/Asian	44 (19.5)
	Other	8 (3.5)
Age, mean (SD)		47.1 (13.7)
Body mass index (kg/m2), mean (SD)		31.1 (6.7)
Smoking history, no. (%)	Never smoked	163 (72.1)
	Ex-smoker	63 (27.9)
Characteristics		
FEV_1_ (L), mean (SD)		2.075 (0.630)
% predicted FEV_1_, mean (SD)		63.4 (11.3)
FEV_1_/FVC (%), mean (SD)		64.4 (10.3)
FEV_1_ reversibility (%), mean (SD)		25.7 (15.6)
ACQ score, mean (SD)		2.46 (0.57)
Mini-AQLQ score, mean (SD)		4.52 (1.04)
Open-label albuterol use^a^, mean (SD)		3.63 (1.62)
Controller: ICS + LABA during the cross-over phase (%)		160 (70.8)
Controller: ICS (low dose^b^) (%)		52 (23.0)
Controller: ICS (medium dose^b^) (%)		157 (69.5)
Controller: ICS (high dose^b^) (%)		17 (7.5)

*ACQ* asthma control questionnaire (7-point scale), *AQLQ* asthma quality of life questionnaire, *FEV*_*1*_ forced expiratory volume in 1 s, *ICS* inhaled corticosteroid, *LABA* long acting β_2_-agonist

^a^Weekly mean puffs albuterol/day

^b^Dose either as ICS monotherapy (low-dose ICS, <320 μg: 9/52 patients [17 %]; medium-dose ICS, >320 – <800 μg: 63/157 patients [40 %]; high-dose ICS, >800 μg: 1/17 patients [6 %]), or ICS component of combination ICS/LABA therapy (low-dose ICS component: 43/52 patients [83 %]; medium-dose ICS component: 94/157 patients [60 %]; high-dose ICS component: 16/17 patients [94 %])

**Fig. 2:**
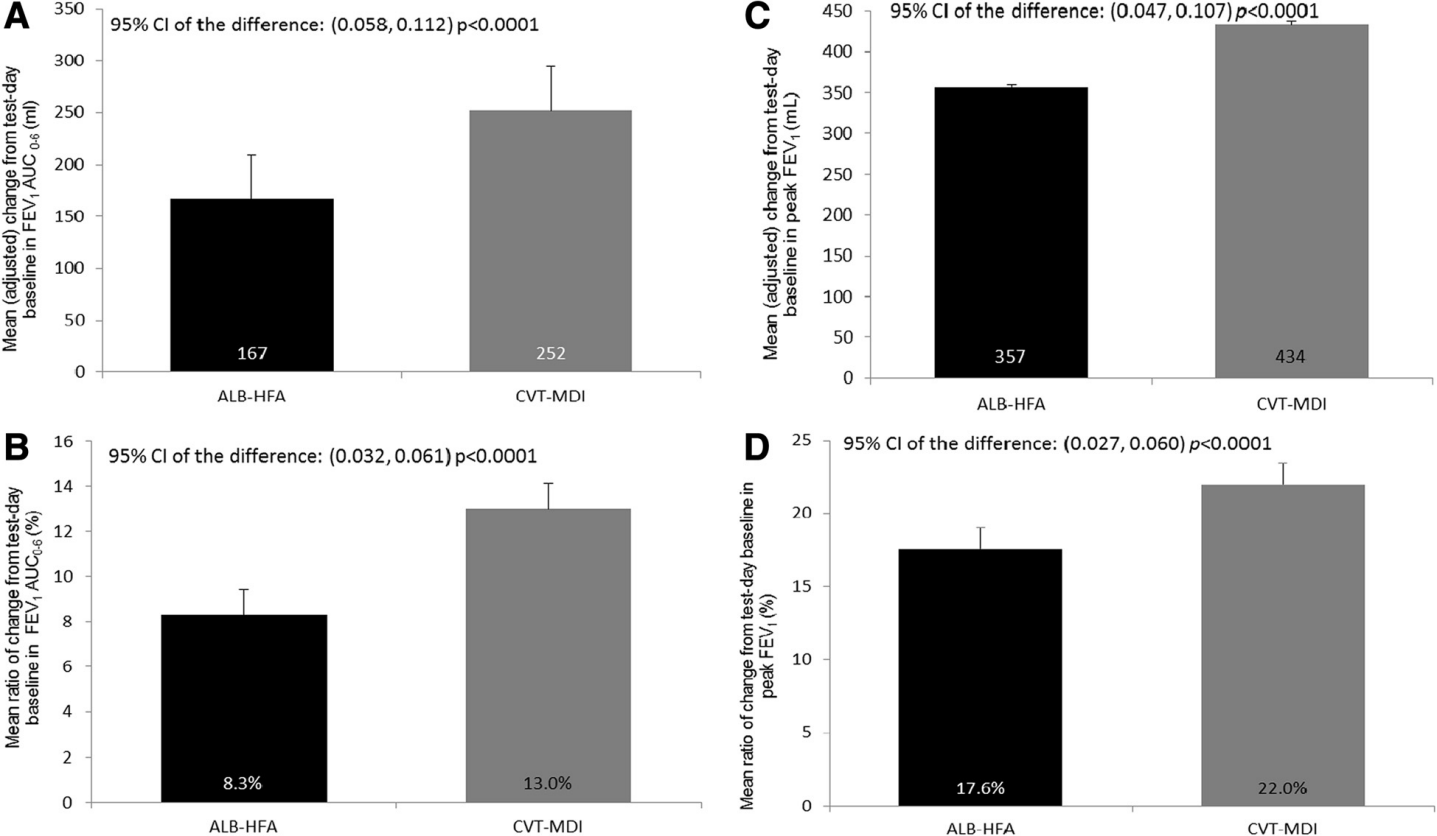
Change from test-day baseline (SE) in FEV_1_ AUC_0–6_ and peak FEV_1_ after 4 weeks. Mean (adjusted) change and post-hoc analysis of mean ratio of change from test-day baseline (SE) in FEV_1_ AUC_0–6_ and peak FEV_1_ after 4 weeks using the mixed-effect model repeated measures (MMRM). **a** Mean (adjusted) change from test-day baseline in FEV_1_ AUC 0–6 (ml) (95 % CI of the difference: (0.058, 0.112) *p* <0.0001); **b** Mean ratio of change from test-day baseline in FEV1 AUC0-6 (%) (95 % CI of the difference: (0.032, 0.061) *p* <0.0001); **c** Mean (adjusted) change from test-day baseline in peak FEV_1_ (ml) (95 % CI of the difference: (0.047, 0.107) *p* <0.0001); **d** Mean ratio of change from test-day baseline in peak FEV_1_ (%) (95 % CI of the difference: (0.027, 0.060) *p* <0.0001)

**Fig. 3:**
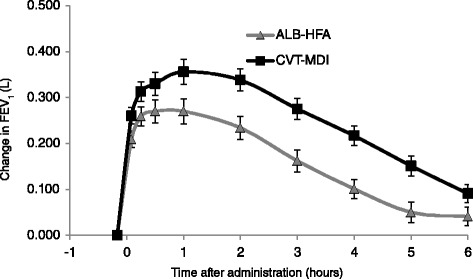
Changes from test-day baseline in FEV_1_. Estimated mean (adjusted) changes from test-day baseline in FEV_1_ at post-dose time-points after 4 weeks using the mixed-effect model repeated measures (MMRM) (day 29; range of difference 50 ml to 115 ml, *p* <0.006 for all comparisons)

At day 29, mean change in FEV_1_ AUC_0–6_ from test-day baseline was 85 ml greater with CVT-MDI versus ALB-HFA (252 ml vs. 167 ml, *p* <0.0001) (Fig. 2a). In a post-hoc analysis, at day 29, the mean ratio of FEV_1_ AUC_0–6_ response to test-day baseline FEV_1_ was 13 % for CVT-MDI and 8.3 % for ALB-HFA (*p* <0.0001) (Fig. 2b). The mean test-day baseline at day 29 for FEV_1_ was 219.3 ml for CVT-MDI and 212.6 ml for ALB-HFA. Mean change from test-day baseline in peak FEV_1_ for CVT-MDI was 77 ml greater than ALB-HFA (434 ml vs. 357 ml, *p* <0.0001) (Fig. 2c). In a post-hoc analysis, at day 29, the mean ratio of peak FEV_1_ response to test-day baseline FEV_1_ was 22 % for CVT-MDI and 17.6 % for ALB-HFA (*p* <0.0001) (Fig. 2d). The mean change from test-day baseline in FEV_1_ was greater at all post-dose time-points in those receiving CVT-MDI (Fig. 3).

**Fig. 4:**
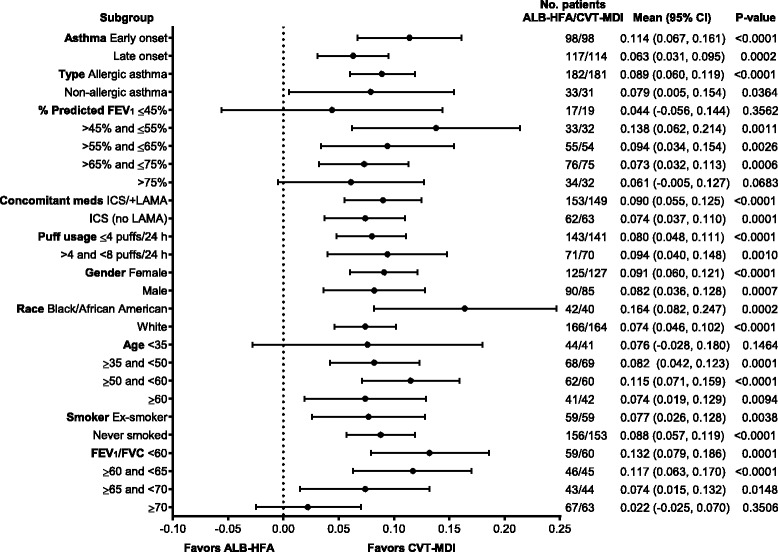
Forest plot of all subgroups for FEV_1_ AUC_0–6_

**Fig. 5:**
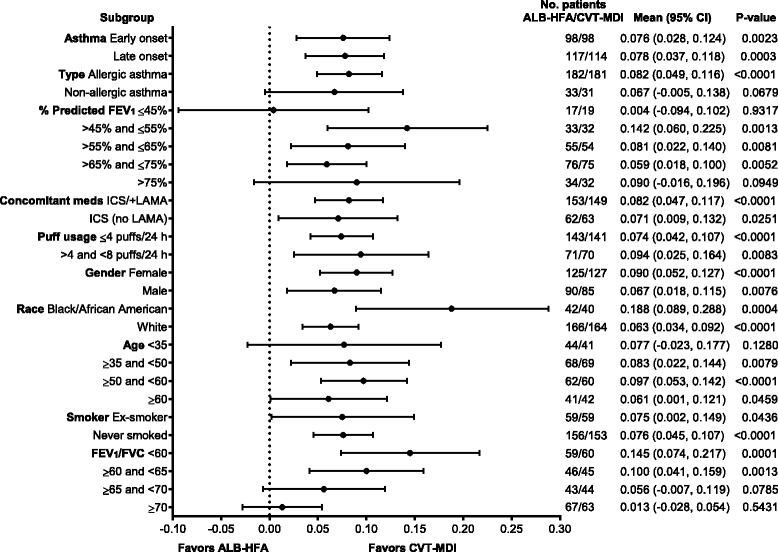
Forest plot of all subgroups for Peak FEV_1_

Greater bronchodilation with CVT-MDI than with ALB-HFA was noted in all subgroups. The subgroup analyses demonstrated that treatment differences were consistent across the subgroups, with no significant treatment by subgroup interactions (Table 2 and Fig. 4 [FEV_1_ AUC_0–6_] and Table 3 and Fig. 5 [peak FEV_1_]). The single exception was a marginally significant treatment by subgroup interaction for the subgroup of onset of asthma for FEV_1_ AUC_0–6_ (*p* = 0.0463); however, this study was not powered to assess whether all subjects with more severe asthma and low lung function (FEV_1_ <45 % predicted) would benefit from the regime.

**Table 2:** FEV_1_ AUC_0−6_ [L] treatment comparisons by subgroups – FAS

Subgroup	Subgroup level	Number of patients ALB-HFA 108/CVT-MDI 18/103	Subgroup by treatment interaction	Difference (CVT-MDI 18/103 – ALB-HFA 108)		
			*p* value*	Mean (SE)	95 % CI	*P* value
Onset of asthma			0.0463			
	Early	98/98		0.114 (0.024)	(0.067, 0.161)	<.0001
	Late	117/114		0.063 (0.016)	(0.031, 0.095)	0.0002
Type of asthma (based on patient history)			0.6444			
	Allergic	182/181		0.089 (0.015)	(0.060, 0.119)	<.0001
	Non−allergic	33/31		0.079 (0.036)	(0.005, 0.154)	0.0364
% Predicted FEV_1_ categories			0.3127			
	≤45 %	17/19		0.044 (0.046)	(−0.056, 0.144)	0.3562
	>45 % and ≤55 %	33/32		0.138 (0.037)	(0.062, 0.214)	0.0011
	>55 % and ≤65 %	55/54		0.094 (0.030)	(0.034, 0.154)	0.0026
	>65 % and ≤75 %	76/75		0.073 (0.020)	(0.032, 0.113)	0.0006
	>75 %	34/32		0.061 (0.033)	(−0.005, 0.127)	0.0683
Concomitant asthma medication usage			0.7610			
	ICS, with LABA	153/149		0.090 (0.018)	(0.055, 0.125)	<.0001
	ICS, without LABA	62/63		0.074 (0.018)	(0.037, 0.110)	0.0001
Puff usage of medication (at baseline, daily average in week prior to visit 3)^a^			0.9938			
	≤4 puffs per 24 h day	143/141		0.080 (0.016)	(0.048, 0.111)	<.0001
	>4 and <8 puffs per 24 h	71/70		0.094 (0.027)	(0.040, 0.148)	0.0010
	≥8 puffs per 24 h day	1/1				
Gender			0.2585			
	Female	125/127	0.2585	0.091 (0.015)	(0.060, 0.121)	<.0001
	Male	90/85		0.082 (0.023)	(0.036, 0.128)	0.0007
Race^b^			0.1311			
	Amer.Ind./Alaska Nat	1/1				
	Asian	2/2				
	Black/African Amer.	42/40		0.164 (0.041)	(0.082, 0.247)	0.0002
	Hawaiian/Pacif. Isle	4/5				
	White	166/164		0.074 (0.014)	(0.046, 0.102)	<.0001
Age category			0.7044			
	<35	44/41		0.076 (0.051)	(−0.028, 0.180)	0.1464
	≥35, <50	68/69		0.082 (0.020)	(0.042, 0.123)	0.0001
	≥50, <60	62/60		0.115 (0.022)	(0.071, 0.159)	<.0001
	≥60	41/42		0.074 (0.027)	(0.019, 0.129)	0.0094
Smoking status			0.9982			
	Ex-smoker	59/59		0.077 (0.026)	(0.026, 0.128)	0.0038
	Never smoked	156/153		0.088 (0.016)	(0.057, 0.119)	<.0001
FEV_1_/FVC [%] pre−BD at Rand			0.1453			
	<60	59/60		0.132 (0.027)	(0.079, 0.186)	<.0001
	≥60, <65	46/45		0.117 (0.026)	(0.063, 0.170)	<.0001
	≥65, <70	43/44		0.074 (0.029)	(0.015, 0.132)	0.0148
	≥70	67/63		0.022 (0.024)	(−0.025, 0.070)	0.3506

*ALB-HFA* albuterol hydrofluoroalkaline, *CVT-MDI* ipratropium bromide/albuterol sulfate metered-dose inhaler, *FAS* full analysis set, *FEV*_*1*_ forced expiratory volume in 1 s, *FVC* forced vital capacity, *ICS* inhaled corticosteroids, *LABA* long-acting β_2_-agonist, *pre-BD at Rand* pre-bronchodilator at randomization

^a^Last category not included in modelling due to insufficient number of patients

^b^Asian, Hawaiian/Pacific Islander and American Indian/Alaska National not included in modelling due to insufficient number of patients

*Unadjusted *p* values

**Table 3:** Peak FEV_1_ [L] treatment comparisons by subgroups − FAS

Subgroup	Subgroup level	Number of patients ALB-HFA 108/CVT-MDI 18/103	Subgroup by treatment interaction	Difference (CVT-MDI 18/103 – ALB-HFA 108)		
			*p* value*	Mean (SE)	95 % CI	*P* value
Onset of asthma			0.7890			
	Early	98/98		0.076 (0.024)	(0.028, 0.124)	0.0023
	Late	117/114		0.078 (0.021)	(0.037, 0.118)	0.0003
Type of asthma (based on patient history)			0.3838			
	Allergic	182/181		0.082 (0.017)	(0.049, 0.116)	<.0001
	Non−allergic	33/31		0.067 (0.035)	(−0.005, 0.138)	0.0679
% Predicted FEV1 categories			0.1378			
	≤45 %	17/19		0.004 (0.046)	(−0.094, 0.102)	0.9317
	>45 % and ≤ 55 %	33/32		0.142 (0.040)	(0.060, 0.225)	0.0013
	>55 % and ≤ 65 %	55/54		0.081 (0.029)	(0.022, 0.140)	0.0081
	>65 % and ≤ 75 %	76/75		0.059 (0.020)	(0.018, 0.100)	0.0052
	> 75 %	34/32		0.090 (0.053)	(−0.016, 0.196)	0.0949
Concomitant asthma medication usage			0.5364			
	ICS, with LABA	153/149		0.082 (0.018)	(0.047, 0.117)	<.0001
	ICS, without LABA	62/63		0.071 (0.031)	(0.009, 0.132)	0.0251
Puff usage of medication (at baseline, daily average in week prior to visit 3)^a^			0.8736			
	≤4 puffs per 24 h day	143/141		0.074 (0.016)	(0.042, 0.107)	<.0001
	>4 and <8 puffs per 24 h	71/70		0.094 (0.035)	(0.025, 0.164)	0.0083
	≥8 puffs per 24 h day	1/1				
Gender			0.1187			
	Female	125/127		0.090 (0.019)	(0.052, 0.127)	<.0001
	Male	90/85		0.067 (0.024)	(0.018, 0.115)	0.0076
Race^b^			0.0980			
	Amer.Ind./Alaska Nat	1/1				
	Asian	2/2				
	Black/African Amer.	42/40		0.188 (0.050)	(0.089, 0.288)	0.0004
	Hawaiian/Pacif. Isle	4/5				
	White	166/164		0.063 (0.015)	(0.034, 0.092)	<.0001
Age category			0.7860			
	<35	44/41		0.077 (0.050)	(−0.023, 0.177)	0.1280
	≥35,< 50	68/69		0.083 (0.031)	(0.022, 0.144)	0.0079
	≥50,< 60	62/60		0.097 (0.022)	(0.053, 0.142)	<.0001
	≥ 60	41/42		0.061 (0.030)	(0.001, 0.121)	0.0459
Smoking status			0.9106			
	Ex−smoker	59/59		0.075 (0.037)	(0.002, 0.149)	0.0436
	Never smoked	156/153		0.076 (0.016)	(0.045, 0.107)	<.0001
FEV1/FVC [%] pre−BD at Rand			0.1852			
	<60	59/60		0.145 (0.036)	(0.074, 0.217)	0.0001
	≥60,< 65	46/45		0.100 (0.029)	(0.041, 0.159)	0.0013
	≥65,< 70	43/44		0.056 (0.031)	(−0.007, 0.119)	0.0785
	≥ 70	67/63		0.013 (0.021)	(−0.028, 0.054)	0.5431

*ALB-HFA* albuterol hydrofluoroalkaline, *CVT-MDI* ipratropium bromide/albuterol sulfate metered-dose inhaler, *FAS* full analysis set, *FEV*_*1*_ forced expiratory volume in 1 s, *FVC* forced vital capacity, *ICS* inhaled corticosteroids, *LABA* long-acting β_2_-agonist, *pre-BD at Rand* pre-bronchodilator at randomization

^a^Last category not included in modelling due to insufficient number of patients

^b^Asian, Hawaiian/Pacific Islander and American Indian/Alaska National not included in modelling due to insufficient number of patients

*Unadjusted *p* values

During each 4-week period of active treatment, there were no significant differences between CVT-MDI and ALB-HFA in number of puffs of study medication used or in number of puffs of open-label (rescue) ALB-HFA used (Table 4). No significant differences were observed in ACQ or mini-AQLQ questionnaire scores, or in the number of nighttime awakenings evaluated at the end of the treatment period (Table 5).

**Table 4:** Analysis of number of puffs of study medication and open-label albuterol used during day and separately at night (mean changes from study baseline^a^) at the end of the 4-week treatment period

	ALB-HFA			CVT-MDI			Difference (CVT-MDI – ALB-HFA)		
Endpoint	n	Mean	SE	N	Mean	SE	Mean	95 % CI	*p* value
Weekly mean^b^ number of AM puffs of study medication used	176	−0.49	0.07	178	−0.53	0.07	−0.04	(−0.17, 0.08)	0.510
Weekly mean^b^ number of PM puffs of study medication used	178	−0.10	0.05	180	−0.12	0.05	−0.02	(−0.12, 0.08)	0.659
Weekly mean^b^ number of AM puffs of open label ALB-HFA used	176	−2.24	0.05	178	−2.28	0.05	−0.04	(−0.14, 0.05)	0.363
Weekly mean^b^ number of PM puffs of open label ALB-HFA used	178	−0.92	0.02	180	−0.93	0.02	−0.01	(−0.06, 0.04)	0.679

*SE* standard error, *95 % CI* 95 % confidence interval, *ALB-HFA* albuterol hydrofluoroalkaline, *CVT-MDI* ipratropium bromide/albuterol sulfate metered-dose inhaler

^a^Mean observed in last week prior to administration of the first dose of the randomized treatment

^b^Weekly mean number of puffs during the fourth week of treatment

**Table 5:** Mean changes from study baseline for Mini-AQLQ, ACQ, and nighttime awakenings for the comparisons of CVT-MDI to ALB-HFA, evaluated at 4 weeks

	ALB-HFA			CVT-MDI			Difference (CVT-MDI – ALB-HFA)		
Endpoint	N	Mean	SE	N	Mean	SE	Mean	(95 % CI)	*p* value
Mini-AQLQ	191	0.15	0.05	193	0.22	0.05	0.06	(−0.03, 0.15)	0.159
ACQ	191	−0.25	0.04	193	−0.25	0.04	0.01	(−0.07, 0.08)	0.828
Nighttime awakenings	178	−0.15	0.02	180	−0.16	0.02	−0.01	(−0.05, 0.04)	0.764

*ACQ* asthma control questionnaire (7-point scale), *ALB-HFA* albuterol hydrofluoroalkaline, *AQLQ* asthma quality of life questionnaire, *CVT-MDI* ipratropium bromide/albuterol sulfate metered-dose inhaler

In a post-hoc analysis, the duration of bronchodilator response at the end of the treatment period was twice as long with CVT-MDI versus ALB-HFA (137.5 min vs. 66.6 min, nominal *p* <0.0001). In another post-hoc analysis, the proportion of responders (peak FEV_1_ response at 4 weeks that was ≥1.15 times the test−day baseline) was significantly greater with CVT-MDI than ALB-HFA: 59.2 % versus 45.6 %, nominal *p* = 0.0014.

Among the 226 patients randomized, 68 (30.1 %) reported at least one AE, with 22.8 % of the CVT-MDI group (mainly due to the higher frequency of cough with CVT-MDI) versus 14 % of patients in the ALB-HFA group reporting an AE. Severe asthma exacerbations (defined in the protocol as worsening of asthma requiring treatment with IV or oral corticosteroids) were reported by seven CVT-MDI patients versus two ALB-HFA patients (none related to study drug). No severe asthma exacerbations were classified as a serious AE or resulted in hospitalization, and no fatal events occurred (Table 6).

**Table 6:** Characteristics of patients with severe asthma exacerbations

	CVT-MDI							ALB-HFA	
	Patient 10868	Patient 11356	Patient 11473	Patient 12410	Patient 12454	Patient 10853	Patient 11752	Patient 11803	Patient 12055
Age (years), gender, race	52, Female, Caucasian	69, Female, Caucasian	64, Female, Hawaiian/PI	36, Female, Caucasian	59, Male, Caucasian	45, Female, Afr Amer	35, Male, Afr Amer	49, Female, Caucasian	74, Female, Caucasian
BMI (kg/m^2^)	33.2	32.5	22.5	48.2	28.6	41.4	N/A	N/A	29.0
Exacerbation - start day, phase	Day 3, Ph 1	Day 2, Ph 1	Day 29, Ph 1	Day 24, Ph 1	Day 15, Ph 1	Day 19, Ph 2	Day 29, Ph 2	Day 11, Ph 2	Day 22, Ph 2
Exacerbation in prior year	None	Yes, 2 months prior	None	Yes, 6 months prior	Yes, 2.5 months prior	None	N/A	Yes, 6 months prior	None
Baseline FEV_1_ % predicted	56.3 %	36.4 %	41.4 %	71.3 %	64.9 %	52.3 %	46.8 %	63.7 %	72.5 %
Background and concomitant asthma medications (daily dose)	Advair 500/100 μg; Alb 180 μg, d/c;	Symbicort 640/18 μg;	Advair 1000/100 μg; Alb 180 μg, d/c;	Advair 250/50 μg; Singulair 10 mg; Zyrtec; Flonase;	Advair 1000/100 μg; Singulair 10 mg;	Advair 1000/100 μg	Advair 1000/100 μg; Singulair 10 mg; Alb 180 μg, d/c; Zyrtec D; Nasonex;	Flovent 440 μg, Combivent 84/480 μg, Alb 180 μg, d/c Flonase	Xopenex 2.5 mg, Pulmicort 360 μg, Alb, d/c
Respiratory infection reported preceding exacerbation	No	No	No	No	No	Yes	Yes	No	No
Use of rescue medication (No. of puffs) 3 days before and day of exacerbation	−3 days: 2	−3 days: 2	−3 days: 4	−3 days: 0	−3 days: 5	−3 days: 2	−3 days: 6	−3 days: 6	−3 days: 4
	−2 days: 4	−2 days: 3	−2 days: 8	−2 days: 2	−2 days: 4	−2 days: 0	−2 days: 8	−2 days:6	−2 days: 4
	−1 day: 2	−1 day: 3	−1 day: 4	−1 day: 2	−1 day: 5	−1 day: 6	−1 day: 6	−1 day: 6	−1 day: 4
	0: 4	0: 6	0: 0	0: 2	0: 7	0: 4	0: N/A	0: 6	0: 6
PEF (L/min) 3 days before and day of exacerbation (best of day)	−3 days: 365	−3 days: 189	−3 days: 155	−3 days: 381	−3 dy: 371	−3 days: 254	−3 days: 388	−3 days: 392	−3 days: 339
	−2 days: 359	−2 days: 160	−2 days: 172	−2 dy: 392	−2 days: 399	−2 days: 227	−2 days: 298	−2 days: 395	−2 days: 334
	−1 day: 353	−1 day: 180	−1 day: 129	−1 day: 426	−1 day: 337	−1 day: 180	−1 day: 307	−1 day: 406	−1 day: 305
	0: 350	0: 181	0: 104	0: 368	0: 353	0: 194	0: 284	0: 374	0: 281
Duration of exacerbation	17 days	9 days	4 days	9 days	7 days	9 days	13 days	81 days	11 days
PEF (L/min) 3 days after end of exacerbation	+1 day: 356	+1 day: 154	N/A	+1 day: 368	+1 day: 492	+1 day: 310	N/A	+1 day: 326	N/A
	+2 days: 311	+2 days: 155		+2 days: 402	+2 days: 486	+2 days: 347		+2 days: 344	
	+3 days: 361	+3 days: 153		+3 days: 384	+3 days: 536	+3 days: 271		+3 days: 314	

*Afr Am* African American, *ALB-HFA* albuterol hydrofluoroalkaline, *Alb* albuterol, *BMI* body mass index, *CVT-MDI* ipratropium bromide/albuterol sulfate metered-dose inhaler, *d/c* discontinued, *FEV*_*1*_ forced expiratory volume in 1 s, *FVC* forced vital capacity, *ICS* inhaled corticosteroids, *LABA* long-acting β_2_-agonist, *PEF* peak expiratory flow, *ph* phase, *PI* pacific islander

## Discussion

This study found a statistically significantly greater bronchodilator effect of CVT-MDI versus ALB-HFA after 4 weeks of “as needed” use of rescue medication, in patients with moderate-to-severe asthma, confirming results from a previous single-dose study [22]. CVT-MDI demonstrated significant improvement in efficacy over ALB-HFA for FEV_1_ AUC_0–6_ response, and peak FEV_1_ response on day 29, indicating maintenance of effect without any evidence of a loss of effect after 4 weeks of as-needed CVT-MDI use. In a post-hoc analysis, the duration of bronchodilator response at the end of the treatment period was twice as long with CVT-MDI compared with ALB-HFA (137.5 min vs. 66.6 min, nominal *p* <0.0001); CVT-MDI has shown consistent benefit in bronchodilation in all subgroups.

As anticipated for medications added for acute symptom relief to maintenance therapy, overall asthma control did not significantly differ between the CVT-MDI and ALB-HFA groups in this short-term, crossover study. The ACQ and mini-AQLQ were developed to measure effects of long-term controller medications, while the investigational medications used in this study were designed for acute symptomatic relief during the 4-week active treatment period. Despite significant differences in lung function, no differences in rescue medication use were observed between the CVT-MDI and ALB-HFA groups. This suggests that the weekly mean number of rescue puffs used per day does not reflect potential differences in lung function improvement during acute symptom relief.

Increasing evidence shows the benefit of anticholinergic agents in moderate-to-severe asthma, including recent studies evaluating tiotropium as add-on therapy in uncontrolled asthma [28, 33–37]. In addition, a Cochrane review of combined inhaled SAMAs and SABAs showed a lower risk of hospital admission and a greater improvement in lung function versus SABAs alone in acute asthma in children [38].

Peters et al. showed that the addition of tiotropium to low-dose ICS resulted in significant improvements in morning and evening PEF, and pre-bronchodilator FEV_1_. The combination of tiotropium and low-dose ICS was comparable to a LABA/ICS combination and was significantly better than doubling the ICS dose [28]. In a separate study of patients with severe uncontrolled asthma despite treatment with at least high-dose ICS plus LABA, the addition of tiotropium significantly improved lung function; however, no significant differences were observed in asthma-related health status or rescue medication use in this crossover and short-term setting, the design of which may have impacted the clinical outcome [33]. In a larger, 48-week study of a similar population of patients, the add-on therapy of tiotropium to high-dose ICS/LABA led to significant increases in lung function and significantly increased the time to first severe asthma exacerbation [34]. Bateman et al. showed that adding tiotropium to medium-dose ICS was non-inferior to salmeterol and superior to placebo in patients with moderate asthma with the B16-Arg/Arg genotype whose asthma was not well controlled with ICS alone [35]. Once-daily tiotropium Respimat® added on to ICS was shown to improve lung function in symptomatic adult [36] and adolescent [37] patients with moderate asthma. These studies support a potentially important therapeutic role for the long-acting anticholinergic tiotropium as maintenance therapy in the treatment of patients with asthma.

In this study, the overall safety profile of CVT-MDI was similar to ALB-HFA. As previously observed with short-acting anticholinergics, patients receiving CVT-MDI reported more cough. The patient population chosen for this study had moderate-to-severe asthma, and was symptomatic despite continuous treatment with ICS with or without LABA and other asthma controller medications, and had ACQ scores ≥1.5. Therefore, in this population with poor asthma control, exacerbations are expected to be more frequent. Although there was a slight excess of patients in the CVT-MDI group (seven CVT-MDI vs. two ALB-HFA patients) who met the protocol-defined criteria for severe asthma exacerbation, no patients were hospitalized, and one patient discontinued on day 29 of the study due to a severe asthma exacerbation. While differences in the number of severe asthma exacerbations between the groups were not significant, exacerbation data was examined in greater detail. An independent review of the severe asthma exacerbations revealed no specific pattern except that five of the seven patients who had severe asthma exacerbations in the CVT-MDI group had a body mass index (BMI) of 32.5–48.2 kg/m^2^. In two of these patients, respiratory infection preceded the exacerbation.

Based on an analysis of the individual eDiary data, there was no increased use or misuse of open label ALB-HFA and/or blinded study medication before the onset of the severe exacerbation in four of the seven CVT-MDI group patients. There was also no evidence of a decrease in the PEF, indicating the onset of worsening of asthma, in four of the seven CVT-MDI group patients.

## Conclusions

These findings are consistent with those from recent studies that demonstrated the value of using the long-acting anticholinergic, tiotropium, as add-on therapy to ICS [39], or as an add-on to ICS plus LABA [34, 40], or compared with doubling the ICS dose [28], in the chronic management of moderate or severe asthma. The results of this study suggest that use of a short-acting anticholinergic bronchodilator in a fixed-dose combination with a SABA has a greater effect on lung function in moderate-to-severe asthma than SABA alone, and should therefore provide better symptomatic relief. In the future, additional studies will be useful to investigate the additive effects of these drugs in patients with all spectra of asthma severity.

These results support the use of a short-acting anticholinergic bronchodilator in a fixed-dose combination with a SABA in asthma, and the need for further clinical trials to determine the role of short- and long-acting anticholinergics (ipratropium and tiotropium, respectively) for managing asthma.

## Availability of data and materials

The synopsis for this study is available at http://trials.boehringer-ingelheim.com/content/dam/internet/opu/clinicaltrial/com_EN/results/1012/1012.57_U10-3568-01-DS.pdf.

The redacted Clinical Study Reports and related clinical documents are available on request, based on a “Document Sharing Agreement”, only for scientific purposes at http://trials.boehringer-ingelheim.com/trial_results/clinical_submissiondocuments_new.html.

Researchers can use the following external platform to request access to raw data from our clinical studies: https://clinicalstudydatarequest.com/.

## Additional file

Additional file 1: Table S1. IRB Approval details from individual centers. (DOC 88 kb)

## Abbreviations

ACQ: asthma control questionnaire
AE: adverse event
ALB-HFA: albuterol hydrofluoroalkaline
AQLQ: asthma quality of life questionnaire
AUC: area under the curve
BMI: body mass index
CFC: chlorofluorocarbon
COPD: chronic obstructive pulmonary disease
CVT: ipratropium bromide/albuterol sulfate
ECG: electrocardiogram
FAS: full analysis set
FEV_1_: forced expiratory volume in 1 second
FVC: forced vital capacity
ICS: inhaled corticosteroid
LABA: long-acting β_2_-agonist
MDI: metered-dose inhaler
MMRM: mixed effect model repeated measures
PEF: peak expiratory flow
SABA: short-acting β_2_-agonist
