# Supplementary material for: Efficacy and safety of ipratropium bromide/albuterol compared with albuterol in patients with moderate-to-severe asthma: a randomized controlled trial
Source: BMC Pulm Med. 2016 Apr 30;16:65. doi: 10.1186/s12890-016-0223-3 (PMC4851785; doi:10.1186/s12890-016-0223-3)
Supplement: Additional file 1: Table S1. — IRB Approval details from individual centers. (DOC 88 kb) [file 12890_2016_223_MOESM1_ESM.doc]

**Supplementary Table 1** IRB Approval details from individual centers.

| Investigator(s) / centre number(s) | IRB or IEC (name/address) | IRB or IEC chairperson | Protocol and/or amendment number(s) | Date of final approval (DD/MMM/YYYY) |
| --- | --- | --- | --- | --- |
| Andrews/101 | Sterling Institutional Review Board  Sterling Independent Services, Inc.  6300 Powers Ferry Road, Suite 600-351  Atlanta, GA 30339 | Sally P. Green, MD | Protocol  Amendment #1  Amendment #2  Amendment #3 | 29-Sep-2008  20-Nov-2008  05-May-2009  27-Jul-2009 |
| Baker /129 | Sterling Institutional Review Board  Sterling Independent Services, Inc.  6300 Powers Ferry Road, Suite 600-351  Atlanta, GA 30339 | Sally P. Green, MD | Protocol  Amendment #1  Amendment #2  Amendment #3 | 21-Oct-2008  20-Nov-2008  05-May-2009  27-Jul-2009 |
| Bensch/ 130 | Sterling Institutional Review Board  Sterling Independent Services, Inc.  6300 Powers Ferry Road, Suite 600-351  Atlanta, GA 30339 | Sally P. Green, MD | Protocol  Amendment #1  Amendment #2  Amendment #3 | 06-Nov-2008  20-Nov-2008  05-May-2009  27-Jul-2009 |
| Berman/ 131 | Sterling Institutional Review Board  Sterling Independent Services, Inc.  6300 Powers Ferry Road, Suite 600-351  Atlanta, GA 30339 | Sally P. Green, MD | Protocol  Amendment #1  Amendment #2  Amendment #3 | 02-Dec-2008  02-Dec-2008  05-May-2009  27-Jul-2009 |
| Bernstein/ 102 | Sterling Institutional Review Board  Sterling Independent Services, Inc.  6300 Powers Ferry Road, Suite 600-351  Atlanta, GA 30339 | Sally P. Green, MD | Protocol  Amendment #1  Amendment #2  Amendment #3 | 23-Sep-2008  20-Nov-2008  05-May-2009  27-Jul-2009 |
| Bleecker/ 128 | Wake Forest University Health Services  Institutional Review Board  The Office of Research and Development  Medical Center Blvd.  Winston-Salem, NC 27157 | Richard Weinberg, MD | Protocol  Amendment #1  Amendment #2  Amendment #3 | 02-Dec-2008  06-Jan-2009  24-Jun-2009  24-Aug-2009 |
| Boscia/ 103 | Sterling Institutional Review Board  Sterling Independent Services, Inc.  6300 Powers Ferry Road, Suite 600-351  Atlanta, GA 30339 | Sally P. Green, MD | Protocol  Amendment #1  Amendment #2  Amendment #3 | 30-Sep-2008  20-Nov-2008  05-May-2009  27-Jul-2009 |
| Brannen/ 104 | Sterling Institutional Review Board  Sterling Independent Services, Inc.  6300 Powers Ferry Road, Suite 600-351  Atlanta, GA 30339 | Sally P. Green, MD | Protocol  Amendment #1  Amendment #2  Amendment #3 | 18-Sep-2008  20-Nov-2008  05-May-2009  27-Jul-2009 |
| Casale/ 132 | Institutional Review Board  Creighton University  Research Compliance Office  2500 California Plaza  Omaha, NE 68178 | David L. Dworzack, MD | Protocol  Amendment #1  Amendment #2  Amendment #3 | 06-Jan-2009  06-Jan-2009  11-May-2009  04-Aug-2009 |
| Corren/ 145 | Sterling Institutional Review Board  Sterling Independent Services, Inc.  6300 Powers Ferry Road, Suite 600-351  Atlanta, GA 30339 | Sally P. Green, MD | Protocol  Amendment #1  Amendment #2  Amendment #3 | 09-Dec-2008  09-Dec-2008  05-May-2009  27-Jul-2009 |
| Covelli/ 107 | Sterling Institutional Review Board  Sterling Independent Services, Inc.  6300 Powers Ferry Road, Suite 600-351  Atlanta, GA 30339 | Sally P. Green, MD | Protocol  Amendment #1  Amendment #2  Amendment #3 | 25-Sep-2008  20-Nov-2008  05-May-2009  27-Jul-2009 |
| Craig/ 108 | Human Subjects Protection Office, Institutional Review Board  Penn State Milton S. Hershey Medical Center  600 Centerview Drive, MC: A115  Hershey, PA 17033 | Kevin Gleeson, MD | Protocol  Amendment #1  Amendment #2  Amendment #3 | 11-Dec-2008  21-Jan-2009  12-May-2009  30-Jul-2009 |
| Donohue/ 109 | Office of Human Research Ethics  University of North Carolina at Chapel Hill  Medical School Building 52  Mason Farm Road, CB 7097  Chapel Hill, NC 27599-7097 | David Weber, MD | Protocol  Amendment #1  Amendment #2  Amendment #3 | 08-Jan-2009  16-Jan-2009  21-May-2009  23-Oct-2009 |
| Fakih/ 149 | Sterling Institutional Review Board  Sterling Independent Services, Inc.  6300 Powers Ferry Road, Suite 600-351  Atlanta, GA 30339 | Sally P. Green, MD | Protocol  Amendment #1  Amendment #2  Amendment #3 | 27-Jan-2009  27-Jan-2009  05-May-2009  27-Jul-2009 |
| Gawchik/ 111 | Sterling Institutional Review Board  Sterling Independent Services, Inc.  6300 Powers Ferry Road, Suite 600-351  Atlanta, GA 30339 | Sally P. Green, MD | Protocol  Amendment #1  Amendment #2  Amendment #3 | 29-Sep-2008  20-Nov-2008  05-May-2009  27-Jul-2009 |
| Greos/ 134 | Sterling Institutional Review Board  Sterling Independent Services, Inc.  6300 Powers Ferry Road, Suite 600-351  Atlanta, GA 30339 | Sally P. Green, MD | Protocol  Amendment #1  Amendment #2  Amendment #3 | 22-Jan-2009  22-Jan-2009  05-May-2009  27-Jul-2009 |
| Kahn/ 112 | Sterling Institutional Review Board  Sterling Independent Services, Inc.  6300 Powers Ferry Road, Suite 600-351  Atlanta, GA 30339 | Sally P. Green, MD | Protocol  Amendment #1  Amendment #2  Amendment #3 | 29-Sep-2008  20-Nov-2008  05-May-2009  Missing *  *site closed 07-27-2009,  no patients enrolled |
| Kaiser/ 113 | Sterling Institutional Review Board  Sterling Independent Services, Inc.  6300 Powers Ferry Road, Suite 600-351  Atlanta, GA 30339 | Sally P. Green, MD | Protocol  Amendment #1  Amendment #2  Amendment #3 | 02-Oct-2008  20-Nov-2008  05-May-2009  27-Jul-2009 |
| Kelsen/ 114 | Temple University  Office for Human Subjects Protections  Institutional Review Board  3400 North Broad Street  Philadelphia, PA 19140 | Michael R. Jacobs, Pharm D | Protocol  Amendment #1  Amendment #2  Amendment #3 | 11-Dec-2008  20-Nov-2008  26-May-2009  10-Sep-2009 |
| Korenblat/ 116 | Sterling Institutional Review Board  Sterling Independent Services, Inc.  6300 Powers Ferry Road, Suite 600-351  Atlanta, GA 30339 | Sally P. Green, MD | Protocol  Amendment #1  Amendment #2  Amendment #3 | 23-Oct-2008  20-Nov-2008  05-May-2009  27-Jul-2009 |
| Koser/ 117 | Sterling Institutional Review Board  Sterling Independent Services, Inc.  6300 Powers Ferry Road, Suite 600-351  Atlanta, GA 30339 | Sally P. Green, MD | Protocol  Amendment #1  Amendment #2  Amendment #3 | 23-Oct-2008  20-Nov-2008  05-May-2009  27-Jul-2009 |
| LaForce/ 118 | Sterling Institutional Review Board  Sterling Independent Services, Inc.  6300 Powers Ferry Road, Suite 600-351  Atlanta, GA 30339 | Sally P. Green, MD | Protocol  Amendment #1  Amendment #2  Amendment #3 | 02-Oct-2008  20-Nov-2008  05-May-2009  27-Jul-2009 |
| Lapidus/ 151 | Sterling Institutional Review Board  Sterling Independent Services, Inc.  6300 Powers Ferry Road, Suite 600-351  Atlanta, GA 30339 | Sally P. Green, MD | Protocol  Amendment #1  Amendment #2  Amendment #3 | 22-Jan-2009  22-Jan-2009  05-May-2009  27-Jul-2009 |
| Mansfield/ 136 | Sterling Institutional Review Board  Sterling Independent Services, Inc.  6300 Powers Ferry Road, Suite 600-351  Atlanta, GA 30339 | Sally P. Green, MD | Protocol  Amendment #1  Amendment #2  Amendment #3 | 07-Oct-2008  20-Nov-2008  05-May-2009  27-Jul-2009 |
| Mathur/ 105 | Western Institutional Review Board  3535 Seventh Avenue  SW-Olympia, WA 98508-5010 | Theodore D. Schultz  Brenda M. Arend | Protocol  Amendment #1  Amendment #2  Amendment #3 | 21-Nov-2008  09-Jan-2009  22-May-2009  21-Aug-2009 |
| Mello/ 126 | Sterling Institutional Review Board  Sterling Independent Services, Inc.  6300 Powers Ferry Road, Suite 600-351  Atlanta, GA 30339 | Sally P. Green, MD | Protocol  Amendment #1  Amendment #2  Amendment #3 | 29-Sep-2008  20-Nov-2008  05-May-2009  27-Jul-2009 |
| Meltzer/ 137 | Sterling Institutional Review Board  Sterling Independent Services, Inc.  6300 Powers Ferry Road, Suite 600-351  Atlanta, GA 30339 | Sally P. Green, MD | Protocol  Amendment #1  Amendment #2  Amendment #3 | 04-Dec-2008  04-Dec-2008  05-May-2009  27-Jul-2009 |
| Miller/ 147 | Sterling Institutional Review Board  Sterling Independent Services, Inc.  6300 Powers Ferry Road, Suite 600-351  Atlanta, GA 30339 | Sally P. Green, MD | Protocol  Amendment #1  Amendment #2  Amendment #3 | 04-Dec-2008  04-Dec-2008  05-May-2009  27-Jul-2009 |
| Moriarity/ 119 | Sterling Institutional Review Board  Sterling Independent Services, Inc.  6300 Powers Ferry Road, Suite 600-351  Atlanta, GA 30339 | Sally P. Green, MD | Protocol  Amendment #1  Amendment #2  Amendment #3 | 16-Oct-2008  20-Nov-2008  05-May-2009  27-Jul-2009 |
| Nayak/ 127 | Sterling Institutional Review Board  Sterling Independent Services, Inc.  6300 Powers Ferry Road, Suite 600-351  Atlanta, GA 30339 | Sally P. Green, MD | Protocol  Amendment #1  Amendment #2  Amendment #3 | 29-Sep-2008  20-Nov-2008  05-May-2009  27-Jul-2009 |
| Noonan/ 138 | Sterling Institutional Review Board  Sterling Independent Services, Inc.  6300 Powers Ferry Road, Suite 600-351  Atlanta, GA 30339 | Sally P. Green, MD | Protocol  Amendment #1  Amendment #2  Amendment #3 | 21-Oct-2008  20-Nov-2008  05-May-2009  27-Jul-2009 |
| Pedinoff/ 139 | Sterling Institutional Review Board  Sterling Independent Services, Inc.  6300 Powers Ferry Road, Suite 600-351  Atlanta, GA 30339 | Sally P. Green, MD | Protocol  Amendment #1  Amendment #2  Amendment #3 | 23-Oct-2008  20-Nov-2008  05-May-2009  27-Jul-2009 |
| Player/ 121 | Cooper Green – Mercy Hospital Institutional Review Board  1515 Sixth Avenue South,  Birmingham, AL 35233 | Martin D. Palmer, MD  Neal M. Miller, MD | Protocol  Amendment #1  Amendment #2  Amendment #3 | 17-Oct-2008  Missing*  Missing*  Missing*  * site closed 22-Jul-2009,  no patients enrolled |
| Pollard/ 140 | Sterling Institutional Review Board  Sterling Independent Services, Inc.  6300 Powers Ferry Road, Suite 600-351  Atlanta, GA 30339 | Sally P. Green, MD | Protocol  Amendment #1  Amendment #2  Amendment #3 | 23-Sep-2008  20-Nov-2008  05-May-2009  27-Jul-2009 |
| Tan/ 124 | Sterling Institutional Review Board  Sterling Independent Services, Inc.  6300 Powers Ferry Road, Suite 600-351  Atlanta, GA 30339 | Sally P. Green, MD | Protocol  Amendment #1  Amendment #2  Amendment #3 | 16-Oct-2008  20-Nov-2008  05-May-2009  27-Jul-2009 |
| Taylor/ 150  (PI until  30-Jun-09)  Baylor/ 150  (PI as of  01-Jul-09) | Sterling Institutional Review Board  Sterling Independent Services, Inc.  6300 Powers Ferry Road, Suite 600-351  Atlanta, GA 30339 | Sally P. Green, MD | Protocol  Amendment #1  Amendment #2  Amendment #3 | 29-Jan-2009  29-Jan-2009  05-May-2009  27-Jul-2009 |
| Tilles/ 141 | Sterling Institutional Review Board  Sterling Independent Services, Inc.  6300 Powers Ferry Road, Suite 600-351  Atlanta, GA 30339 | Sally P. Green, MD | Protocol  Amendment #1  Amendment #2  Amendment #3 | 23-Sep-2008  20-Nov-2008  05-May-2009  27-Jul-2009 |

| Wachtel/ 144 | Sterling Institutional Review Board  Sterling Independent Services, Inc.  6300 Powers Ferry Road, Suite 600-351  Atlanta, GA 30339 | Sally P. Green, MD | Protocol  Amendment #1  Amendment #2  Amendment #3 | 11-Dec-2008  11-Dec-2008  05-May-2009  27-Jul-2009 |
| --- | --- | --- | --- | --- |
| Walker/ 142 | Sterling Institutional Review Board  Sterling Independent Services, Inc.  6300 Powers Ferry Road, Suite 600-351  Atlanta, GA 30339 | Sally P. Green, MD | Protocol  Amendment #1  Amendment #2  Amendment #3 | 23-Sep-2008  11-Dec-2008  05-May-2009  27-Jul-2009 |
| Wanderer/ 146 | Sterling Institutional Review Board  Sterling Independent Services, Inc.  6300 Powers Ferry Road, Suite 600-351  Atlanta, GA 30339 | Sally P. Green, MD | Protocol  Amendment #1  Amendment #2  Amendment #3 | 16-Dec-2008  16-Dec-2008  05-May-2009  27-Jul-2009 |
| Webster/ 148 | Sterling Institutional Review Board  Sterling Independent Services, Inc.  6300 Powers Ferry Road, Suite 600-351  Atlanta, GA 30339 | Sally P. Green, MD | Protocol  Amendment #1  Amendment #2  Amendment #3 | 23-Dec-2008  23-Dec-2008  05-May-2009  27-Jul-2009 |
| Wise/ 143 | Office of Human Subjects Research  JHM Institutional Review Board 5  1620 McElderry Street  Reed Hall, Suite B-130  Baltimore, MD 21205-1911 | Gary Briefel, MD | Protocol  Amendment #1  Amendment #2  Amendment #3 | 20-Jan-2009  02-Mar-2009  06-Jul-2009  21-Sep-2009 |
